# Changing and challenging times for Canadian kidney health and disease research

**DOI:** 10.1186/s40697-015-0085-2

**Published:** 2015-12-01

**Authors:** C. R. J. Kennedy

**Affiliations:** Kidney Research Centre, Chronic Disease Program, Department of Medicine, Ottawa Hospital Research Institute, Ottawa, Ontario Canada; Department of Cellular and Molecular Medicine, Faculty of Medicine, University of Ottawa, Ontario, Canada; Ottawa Hospital Research Institute, Ottawa Hospital and University of Ottawa, 451 Smyth Road, Room 2515, Ottawa, Ontario K1H 8M5 Canada

“It was the best of times, it was the worst of times, it was the age of wisdom, it was the age of foolishness, it was the epoch of belief, it was the epoch of incredulity, it was the season of Light, it was the season of Darkness, it was the spring of hope, it was the winter of despair, we had everything before us, we had nothing before us.” Charles Dickens, A Tale of Two Cities

## The issues

In many ways, the Canadian renal researcher lives in privileged times. Never before has she or he enjoyed such remarkable access to the tools of the trade. Unprecedented advances in biotechnology, genomics, molecular biology, regenerative medicine, tissue banking, and computer databases along with the implementation of numerous infrastructure projects on a scale unparalleled in our history have together luxuriously equipped our scientific community. We stand poised for breakthroughs that will significantly impact the renal health of Canadians both tomorrow and well into the future. Thanks to initiatives such as the federally funded Canada Foundation for Innovation along with its provincial and local partners, it is not uncommon for both basic and clinician scientists specializing in nephrology to be employing state-of-the-art equipment, housed in dozens of newly minted laboratories and research buildings across our country. Moreover, training programs such as the Kidney Foundation of Canada (KFoC)/Canadian Institutes of Health Research (CIHR) supported Kidney Research Scientist Core Education and National Training Program (KRESCENT) initiative have fostered the development of the next generation of investigators, each eager to contribute to the advancement of knowledge.

However, the times wherein the Canadian renal researcher lives are also defined by enormous challenges. Despite the wealth of research infrastructure and an abundance of well-trained investigators, as in the USA, our national research endeavor seems to have been designed however, inadvertently, in anticipation of unlimited growth. Over the past 15 years, universities, hospitals, and research institutes have continued to hire into new positions in order to fill expanding wet and dry lab space to capacity. Despite this increase in the hiring of principal investigators, our system continues to train far more graduate students and postdoctoral fellows than there are positions in academia, industry, and government. Competition for new investigator spots is fierce, forcing many individuals to remain in training positions during their most creative years until either obtaining their first independent position or leaving research for other career options. These issues have imposed stress upon the system and together with an unchanging pool of funds available to investigators, have driven down funding success for many individual research operations.

Not long ago, CIHR funding levels hovered at the 20–27 % range; on some peer-review committees, this was as high as 40 %, allowing for most applications ranked in the excellent range (i.e., ≥4.0 on the CIHR scale) to be approved. Recent open operating grant competitions have seen application pressure steadily increase, placing greater burdens on both reviewers and their committees. This has created a vicious cycle where highly ranked proposals line up in the queue, often requiring several resubmissions before being funded, if at all. With the declining success rates for CIHR applications, more applicants have sought funding from other agencies, similarly driving up application pressures and decreasing success rates. The net effect has been that funding rates with most sources are now at historic lows.

Predictable, long-term funding for research projects has proved increasingly difficult to sustain as these success rates drop, often forcing labs to lose key staff in order to endure down cycles. Moreover, the recent devaluation of the Canadian versus American dollar has further destabilized matters since many consumables are sourced from the USA. Meanwhile, the capacity for extramural funding agencies such as the Kidney Foundation of Canada, a longtime supporter of renal research, has remained limited over the past 15 years. Today’s CAD$50,000 per annum, KFoC biomedical research grant has the purchasing power equivalent to CAD$36,000 in the year 2000. The KFoC is not the lone example however; fundraising efforts, endowments and industry-based “no-strings-attached” money have all suffered since the “Great Recession” of 2008. Despite widespread cuts to most departments, the federal government has not reduced the level of funding to the CIHR. Unfortunately, the relatively stagnant pool of research dollars has struggled to keep pace with inflation in biomedical research. The net result of these economic realities, coupled with an expanding pool of investigators is that today’s renal scientist is spending more of their time writing a greater number of grant applications in a far more competitive environment than their peers a generation ago—all at the expense of carrying out actual research.

## New funding structures

At the same time, the CIHR recently introduced significant broad-based changes to how it evaluates and funds scientific research. At the risk of understating the impact, the revamped open suites programs (Foundation and Project schemes) has generated a broad spectrum of opinions among Canada’s scientific research community. A formal critique of the new format would be premature as the data have not yet been assembled, disseminated, and interpreted. Moreover, this nascent system remains fluid as it undergoes adjustments and amendments in response to internal assessments as well as both applicant and reviewer feedback. For example, it was welcome news that the CIHR recently reversed its decision to only allow applicants a single opportunity per calendar year to apply for project grants. If this position had been maintained, researchers’ ability to acquire funding in a timely manner would have been severely limited.

For the renal research community, the new formats for application and review need to be disseminated so that kidney disease research maintains an appropriate level on the Canadian science scene. Importantly, given the implementation of the college of reviewers, it is essential that those within the renal research community offer their full participation in the review process. The best minds in kidney disease research should take their seats at the (virtual) review table so that applications are evaluated by the most appropriate experts who are intimately familiar with our field. The same holds true for the updated KFoC Biomedical Research Grant application format and its review committee. Importantly, the question of whether these new directions will adequately address the realities facing our collective research endeavor remains unanswered.

## Radical options?

Even with such dramatic changes to the funding mechanisms at CIHR, are other options possible? Our American colleagues are beginning to entertain far more radical changes to their system. A grass roots movement is emerging in the USA aimed at opening a dialogue between scientists and institutions to challenge existing research paradigms. The ideas were presented in two recent essays [1, 2]. The authors summarize the current plight of scientific research in the US system and propose a number of solutions. Perhaps surprisingly, a demand for more money was not suggested, despite the recently announced proposals by the House of Representatives and Senate panels that would increase the NIH budget by CAD$1.1–2 billion per year. They suggest training fewer postdoctoral fellows and PhDs, with some of the former being transitioned within labs to the so-called super-doc positions. The idea is that these highly trained individuals would graduate to staff-scientists, capable of preserving the intra-laboratory memory and offering high productivity levels [3]. There is even the idea that funding agencies could mandate universities to direct a portion of their overhead payments towards creating more of these types of staff-scientist positions. Another proposal is to cease supporting graduate students from NIH research grants—relying instead on institutional training grants and external scholarships.

Meanwhile the authors recommend that funding agencies should be wary of “overfunding” labs since the law of diminishing returns inevitably kicks in past a certain threshold of research dollars. Such changes would reduce the number of “megalabs” while encouraging smaller more sustainable operations—where principal investigators would spend less time chasing research dollars to fund operations. Moreover, the authors take aim at the perceived trend towards translational research. They state that “Overvaluing translational research is detracting from an equivalent appreciation of fundamental research of broad applicability, without obvious connections to medicine.” [1].

Lastly, the authors suggest that agencies should reward projects that focus on originality and risk-taking to discourage predictable, incremental research—which inevitably arises when funding is difficult to obtain, and research results are expected to be translated in the short term to bear immediate fruit for society. What is often lost is the value of the long view. Indeed, we value instant gratification and rapid return on investment. In the past, many of Europe’s great cathedrals required lengthy construction periods. The original architects and builders were almost never worshipped in the finished product. They held a long view of their work. Can the same be said for the current Canadian research environment? While tremendous value should be placed on carrying out work that provides advances that are implemented in the short term for the benefit of Canadians (e.g., Strategy for Patient Oriented Research (SPOR), research aimed at informing clinical practice guidelines, etc.), there are some who feel that the balance in research structure is tipping away from investigation that holds the long view. Thus, scientists craft safer research proposals that yield predictable findings thereby advancing their field invariably in incremental steps.

Have you heard of Janelia Research Campus? It might be the penultimate “long view” research institution. Originally named Janelia Farm, this is a free-standing research campus of the Howard Hughes Medical Institute opened in 2006 (https://www.janelia.org/). It is decidedly atypical in its structure and its operation philosophy. It was originated to address some of the major issues facing the traditional model of scientific research—namely an aversion to high-risk endeavors and an inability to rapidly adapt research programs to embrace opportunities that emerge with new discoveries [4]. Researchers at Janelia Research Campus do not write grants, and publication in peer-reviewed journals is not viewed as the ultimate goal (although its scientists routinely publish in very high-impact journals). In contrast to the traditional research institute model where groups of scientists with a wide variety of research interests are assembled and divided into faculties, departments, and programs, the developers of Janelia Farm limited the field to two initial areas of scientific focus. The first was the identification of principles that govern how information is processed by neuronal circuits, using genetic model systems in conjunction with imaging, electrophysiological, and computational methods. The second was the development of imaging approaches and computational methods for image analysis. For the founders of Janelia, these areas of research provided highly focused yet challenging project themes that would benefit from “patient, generous funding in an environment that fosters free-flowing dialogue, critique, and creative problem solving across multiple disciplines—an environment not easily created in current research institutions” [4]. Taken together, the goal was to assemble the best minds in these areas, let them “live science” together on a daily basis, to not bother them too much for a fairly long period of time, and see what they come up with. Today, Janelia Research Campus continues to build its research legacy, yet in accordance with its long view, its impact will be judged by future generations [5].

While radically transforming existing research structures along these lines sounds utopic and perhaps even impractical for Canadian renal science, both the recent dialogue about restructuring the research paradigm in the USA and the Janelia Research Campus experiment are instructive. For example, given our limited size and capacity in Canada, would it be useful to ask fewer research questions—to increase focus and enhance collaboration among Canadian renal researchers? Similarly, should we identify the key strengths of the Canadian nephrology research community and accordingly, expand the number of integrative teams to tackle these key questions? Or, should we consider downsizing labs and consolidating resources within existing institutions—supporting more shared staff-scientists, and fewer students and postdocs? And lastly, in terms of engaging with our funding agencies: we need to participate fully in the new CIHR review process as well as that of the KFoC in order to foster and support Canadian research into kidney health and disease.

While the Dickens quote at the beginning of this editorial may be viewed as hyperbole, in many ways, these are indeed the best of times and the worst of times for the wider renal research community in Canada. While opportunities remain, challenges are many, for which we await creative solutions.
